# Há um Fôlego Extra para o Uso de Inibidores da Glicoproteína IIb/IIIa em Mulheres Diabéticas Idosas com Infarto do Miocárdio com Elevação de ST ou Estamos em uma Situação de Risco?

**DOI:** 10.36660/abc.20200494

**Published:** 2021-02-19

**Authors:** Ana Teresa Timóteo

**Affiliations:** 1 Santa Marta HospitalCentro Hospitalar Universitário Lisboa CentralLisboaPortugalSanta Marta Hospital, Centro Hospitalar Universitário Lisboa Central, Lisboa - Portugal; 2 Universidade Nova de LisboaFaculdade de Ciências Médicas de LisboaLisboaPortugalUniversidade Nova de Lisboa, Faculdade de Ciências Médicas de Lisboa, Lisboa - Portugal

**Keywords:** Agregação Plaquetária, Inibidores da Glicoproteína IIb/IIIa, Idoso, Mulheres, Diabetes Mellitus, Infarto do Miocárdio, Hemorragia

O padrão de tratamento de pacientes submetidos à intervenção coronária percutânea (ICP) primária é a terapia antiplaquetária dupla (TAPD), com uma combinação de aspirina e um inibidor P2Y12.^1^ Prasugrel e ticagrelor são os inibidores P2Y12 preferidos porque têm início de ação mais rápido, maior potência e são superiores ao clopidogrel em relação aos desfechos clínicos.^1^ Eles devem ser mantidos por 12 meses, a menos que haja contraindicações, como risco excessivo de sangramento.^1^ A escolha do tratamento deve ser uma decisão equilibrada, considerando os riscos de isquemia e sangramento. A maioria dos estudos que avaliam os inibidores da glicoproteína (Gp) IIb/ IIIa em pacientes com infarto agudo do miocárdio com supradesnivelamento do segmento ST (IAMCSST) tratados com ICP primária são anteriores à era do pré-tratamento oral de rotina com TAPD, particularmente no contexto de uso de inibidores de plaquetas orais potentes. Naquela época, eles demonstraram uma redução na incidência de eventos isquêmicos, mas à custa de um aumento consistente de sangramentos maiores.^2^ Atualmente, não há evidências convincentes de um benefício adicional do uso rotineiro de uma estratégia de uso de Gp IIb/IIIa em pacientes com ICP primária que recebem tratamento com TAPD, particularmente com ticagrelor.^2^ O uso de inibidores de Gp IIb/IIIa deve ser considerado como terapia de resgate em caso de evidência angiográfica de um grande trombo, fenômenos de *slow *ou *no-reflow*, e outras complicações trombóticas, embora essa estratégia não tenha sido abordada em ensaios clínicos randomizados.^2^ Além disso, a administração intracoronária não é superior ao seu uso intravenoso.^3^

Pacientes idosos apresentam alto risco de sangramento e outras complicações decorrentes de terapias agudas, não apenas por causa da idade, mas por apresentarem com maior frequência disfunção renal e mais comorbidades.^2^ Diabetes também é uma comorbidade frequente em pacientes com IAMCSST. Pacientes diabéticos apresentam doença aterosclerótica mais difusa e maior risco de morte e complicações, incluindo revascularização repetida após ICP.^2^ De fato, pacientes diabéticos que sofreram infarto do miocárdio têm um pior prognóstico, e a presença de diabetes aumenta o risco de qualquer evento cardiovascular, como demonstrado em diversos estudos anteriores de tratamento da síndrome coronariana aguda.^1,2,4^ Entretanto, no contexto atual de uso de inibidores P2Y12 orais, não há indicação de que a farmacoterapia antitrombótica deva ser diferente entre pacientes diabéticos e aqueles sem diabetes submetidos à revascularização.^1,2^

Na presente edição desta revista, um grupo chinês investigou o possível benefício da terapia antiplaquetária tripla (TAPT) com aspirina, ticagrelor e tirofibana, em pacientes diabéticas idosas, em comparação com TAPD.^5^ Eles estudaram 162 mulheres idosas e diabéticas separadamente em dois grupos de acordo com o escore CRUSADE. O grupo com menor escore CRUSADE (<30) recebeu TAPT, assim como um grupo controle de 97 pacientes idosos e diabéticos do sexo masculino, também com baixo escore CRUSADE. O grupo de pacientes do sexo feminino com pontuação CRUSADE alta recebeu apenas TAPD. Em geral, as mulheres apresentaram mais complicações isquêmicas e hemorrágicas. Ao comparar homens e mulheres com escore CRUSADE baixo e que receberam TAPT, apesar do tratamento semelhante, as mulheres apresentaram mais reinfarto, trombose de stent, choque cardiogênico e mortalidade em 30 dias, mas também apresentaram mais sangramento moderado e grave. Comparando apenas os grupos femininos, o grupo que recebeu TAPD teve menor recuperação em termos de escore TIMI e classificação de perfusão miocárdica pelo escore TIMI após a ICP, mais trombose de stent, choque cardiogênico e mortalidade em 30 dias, mas menos sangramento moderado / grave. Os autores concluíram que a estratégia de TAPT em mulheres idosas diabéticas com IAMCSST apresentou menos eventos cardiovasculares no seguimento de 30 dias, mas apresentou mais complicações hemorrágicas.

No entanto, algumas observações e limitações devem ser destacadas. Em primeiro lugar, a média de idade dos pacientes em cada grupo não é de fato equivalente à “idoso” como poderíamos defini-la. A média de idade está em torno de 65 anos em todos os grupos e “idoso” foi definido como acima de 60 anos. Por esse motivo, a TAPT foi testada em pacientes relativamente jovens. Assim, as conclusões não podem ser tiradas para pacientes verdadeiramente idosos, os quais podem ser considerados como aqueles com mais de 75 anos de idade. Em segundo lugar, a inclusão foi realizada durante seis anos e uma média de 43 pacientes/ano foram incluídos. Podemos suspeitar que o centro tem um volume moderado, ou a inclusão não foi consecutiva e um número significativo de pacientes não foi incluído. Por essa razão, a amostra é relativamente pequena e o poder estatístico não é adequado. Em terceiro lugar, em estudos com terapias antitrombóticas, é importante fornecer resultados adicionais, particularmente na forma de desfechos compostos abrangendo eventos isquêmicos e hemorrágicos, como o Benefício Clínico Líquido ou Eventos Clínicos Adversos Líquidos.

Esta é a melhor maneira de definir claramente se os benefícios superam os riscos e problemas de segurança. Em quarto lugar, o índice de massa corporal (IMC) médio é alto, com um IMC médio de cerca de 30 em todos os grupos. Podemos supor que pacientes com peso corporal normal ou baixo, um importante fator de risco para sangramento, não foram incluídos. Finalmente, a análise multivariável deveria ter sido realizada para confirmar o benefício da TAPT sobre a TAPD em pacientes idosas e diabéticas. Há algumas diferenças nas características da linha de base que podem ter um impacto significativo no desfecho que deveriam ter sido ajustadas. Apesar de todas as limitações apontadas, existem, no entanto, dois pontos importantes que podemos observar com base nos resultados apresentados. Ao contrário do que se pensa, não observamos diferença nos intervalos de tempo em mulheres idosas diabéticas, quando comparadas aos homens, e a anatomia coronariana também foi semelhante.

Em conclusão, o presente estudo não fornece evidências suficientes para alterar a prática clínica usual em pacientes idosas diabéticas com IAMCSST. Muitas perguntas não foram respondidas adequadamente e o benefício adicional do uso rotineiro de Gp IIb/IIIa juntamente com TAPD potente em ICP primária neste subgrupo de pacientes não foi incontestavelmente demonstrado.
