# Supplementary material for: Há um Fôlego Extra para o Uso de Inibidores da Glicoproteína IIb/IIIa em Mulheres Diabéticas Idosas com Infarto do Miocárdio com Elevação de ST ou Estamos em uma Situação de Risco?
Source: Arq Bras Cardiol. 2021 Feb 19;116(2):236–7. [Article in Portuguese] doi: 10.36660/abc.20200494 (PMC7909978; doi:10.36660/abc.20200494)
Supplement: Translation

# Translation: Is There a Second Wind for Glycoprotein IIb/IIIa Inhibitors in Elderly Diabetic Females with ST-Elevation Myocardial Infarction, or are We on Thin Ice?

**Authors:** Ana Teresa Timóteo

**Affiliations:** 1 PortugalSanta Marta Hospital, Centro Hospitalar Universitário Lisboa Central, Lisboa - Portugal; 2 PortugalUniversidade Nova de Lisboa, Faculdade de Ciências Médicas de Lisboa, Lisboa - Portugal

**Keywords:** Platelet Agregation, Glycoprotein IIb/IIIa inhibitors, Elderly, Women, Diabetes Mellitus, Myocardial Infarction, Hemorrhage

The standard of care in patients undergoing primary percutaneous coronary intervention (PCI) is double antiplatelet therapy (DAPT), with a combination of aspirin and a P2Y12 inhibitor.^1^ Prasugrel and ticagrelor are the preferred P2Y12 inhibitors because they have a more rapid onset of action, greater potency, and are superior to clopidogrel in terms of clinical outcomes.^1^ They should be maintained over 12 months unless there are contraindications, such as excessive risk of bleeding.^1^ The choice of treatment should be a balanced decision, considering the ischemic and bleeding risks. Most of the trials evaluating glycoprotein (Gp) IIb/IIIa inhibitors in ST-elevation myocardial infarction (STEMI) patients treated with primary PCI pre-date the era of routine oral DAPT pre-treatment, particularly in the setting of potent oral platelet inhibitors. At that time, they demonstrated a reduction in the incidence of ischemic events, but at the expense of a consistent increase in major bleeding.^2^ Presently, there is no compelling evidence for an additional benefit of the routine use of a Gp IIb/IIIa strategy in primary PCI patients that receive DAPT treatment, particularly with ticagrelor.^2^ The use of Gp IIb/IIIa inhibitors should be considered for bailout therapy in the event of angiographic evidence of a large thrombus, slow- or no-reflow, and other thrombotic complications, although this strategy has not been addressed in randomized controlled trials.^2^ Also, intracoronary administration is not superior to its intravenous use.^3^

Elderly patients are at high-risk of bleeding and other complications from acute therapies, not only because of their age, but because they have more often renal dysfunction and more co-morbidities.^2^ Diabetes is also a frequent comorbidity in STEMI patients. Diabetic patients have more diffuse atherosclerotic disease and are at higher risk of death and complications, including repeated revascularization after PCI.^2^ In fact, diabetic patients who have suffered a myocardial infarction have a worse prognosis, and the presence of diabetes amplifies the risk of any cardiovascular event, as shown in many previous studies of acute coronary syndrome treatment.^1,2,4^ However, in the current context of the use of oral P2Y12 inhibitors, there is no indication that antithrombotic pharmacotherapy should differ between diabetics and patients without diabetes undergoing revascularization.^1,2^

In the present number of this journal, a Chinese group investigated the possible benefit of triple anti-platelet therapy (TAPT) with aspirin, ticagrelor and tirofiban, in elderly female diabetic patients compared to DAPT.^5^ They studied 162 elderly and diabetic female patients separately in two groups according to the CRUSADE score. The group with a lower CRUSADE score (< 30) received TAPT, as well as a control group of 97 elderly and diabetic males, also with low CRUSADE score. The female group with high CRUSADE score received only DAPT. In general, women had more ischemic and hemorrhagic complications. When comparing males and females with low CRUSADE score and receiving TAPT, despite similar treatment, women had more re-infarction, stent thrombosis, cardiogenic shock and 30-day mortality, but also more moderate and severe bleeding. Comparing only the female groups, the group that received DAPT had less recovery in terms of TIMI grading and TIMI myocardial perfusion grading after PCI, more stent thrombosis, cardiogenic shock and 30-day mortality, but less moderate / severe bleeding. The authors concluded that a TAPT strategy in elderly diabetic women with STEMI showed less cardiovascular events at 30-day follow-up but more hemorrhagic complications.

However, some remarks and limitations should be highlighted. Firstly, the mean age of the patients in each group is in fact not “elderly” as we might define it. Mean age is around 65 years in all groups and “elderly” was defined as above 60 years. For that reason, TAPT was tested in relatively young patients. Thus, the conclusions cannot be drawn for truly elderly patients, who might be considered as those above 75 years of age. Secondly, inclusion was performed for six years and an average of 43 patients/ year were included. We can suspect that the centre has a moderate volume, or inclusion was not consecutive, and a significant number of patients were not included. For that reason, the sample is relatively small and statistical power is not adequate. Thirdly, in studies with anti-thrombotic therapies, it is important to provide additional results, particularly in the form of composite outcomes encompassing ischemic and bleeding events, such as Net Clinical Benefit or Net Adverse Clinical Events. This is a better way to clearly define if the benefits outweigh the risks and safety problems. Fourthly, mean body mass index (BMI) is high, with a mean BMI of around 30 in all groups. We can assume that patients with normal or low body weight, an important risk factor for bleeding, were not included. Finally, multivariable analysis should have been performed to confirm the benefit of TAPT over DAPT in elderly and diabetic female patients. There are some differences in baseline characteristics that might have a significant impact on the outcome that should have been adjusted. Despite all the highlighted limitations, there are, however, two important points that we can observe based on the presented results. Contrary to general belief, we did not observe any difference in time delays in elderly diabetic women, when compared to men and coronary anatomy was also similar.

In conclusion, the present study does not give enough evidence to change the usual clinical practice in STEMI elderly diabetic female patients. Many questions were not properly addressed and the additional benefit of routine use of Gp IIb/IIIa together with potent DAPT in primary PCI in this sub-group of patients was not undoubtedly demonstrated.
